# The commercial harvest of ice-associated seals in the Sea of Okhotsk, 1972-1994

**DOI:** 10.1371/journal.pone.0182725

**Published:** 2017-08-10

**Authors:** Irina S. Trukhanova, Aleksey I. Grachev, Aleksandr G. Somov, Vladimir N. Burkanov, Kristin L. Laidre, Peter L. Boveng

**Affiliations:** 1 Polar Science Center, Applied Physics Laboratory, University of Washington, Seattle, Washington, United States of America; 2 Marine Mammal Laboratory, Alaska Fisheries Science Center, National Oceanic and Atmospheric Administration, Seattle, Washington, United States of America; 3 Laboratory of coastal resources, Magadan Research Institute of Fisheries and Oceanography (MagadanNIRO), Magadan, Russia; 4 Laboratory of marine mammals, Russian Federal Research Institute of Fisheries and Oceanography (VNIRO), Moscow, Russia; 5 Kamchatka Branch of the Pacific Geographical Institute Far East Branch of Russian Academy of Sciences, Petropavlovsk-Kamchatskiy, Russia; U.S. Geological Survey, UNITED STATES

## Abstract

Sealing log books from 75 out of 79 commercial harvest cruises carried out between 1972 and 1994 in the Sea of Okhotsk, Russia, were analyzed to describe spatial and temporal allocation of ice-associated seal harvest effort, species composition of catches, total harvest rates, and related parameters for species including ringed (*Pusa hispida*), ribbon (*Histriophoca fasciata*), bearded (*Erignathus barbatus*) and spotted (*Phoca largha*) seal. Variations in catch per unit effort were explored in relation to year, sea ice conditions, day of the year, and geographic location. In most years, the harvest was predominantly represented by ringed seals (mean = 0.43, range 0.25–0.67), followed by ribbon (mean = 0.31, range 0.15–0.43), spotted (mean = 0.19, range 0.11–0.35) and bearded seals (mean = 0.07, range 0.03–0.14). The struck-and-lost percentages were as high as 30–35% for ringed, bearded and spotted seals and 15–20% for ribbon seals. Catch per unit effort (number of seals/skiff*day) for ringed, ribbon, and spotted seals had a similar seasonal pattern with a distinct spike in catches for spotted seals in the first week of May, for ribbon seals in the last week of May, and for ringed seals in the second week of June. Catches of bearded seals showed a less pronounced temporal structure with a gradual increase toward the end of the harvest season in the majority of years. Spatial distribution of harvest effort followed closely with seal distribution obtained from aerial surveys. These data could be used as a source of information on seal herd location throughout the breeding and molting seasons and for more complex demographic or life-table models. We did not find any evidence of the decline of catch per unit effort over the study period. Timely introduction of state regulations and efficient harvest management apparently prevented severe depletion of ice-associated seal populations in the Sea of Okhotsk during the periods of their intense exploitation.

## Introduction

The Sea of Okhotsk is inhabited by four ice-associated seal species: ringed (*Pusa hispida*), ribbon (*Histriophoca fasciata*), bearded (*Erignathus barbatus*), and spotted (*Phoca largha*) seal. All four species have a long history of exploitation including periods of aboriginal and commercial harvests, shore- or vessel-based harvests, and unlimited or state-regulated harvests [1–7]. The commercial harvest conducted from 1931 to 1994 provided extensive data on seal reproductive biology, demographic structure of the populations, habitat preferences, and seasonal distribution [3, 6, 8]. The participation of professional biologists aboard sealing vessels, as well as a centralized system of catch reporting used during the Soviet era, allowed for a large portion of catches to be thoroughly examined and a large amount of data to be published in the Russian scientific literature (see e.g. [7, 8]).

However, there remain many internal reports stored in resource institutions throughout the Russian Federation that contain data that could provide detailed insights on historical ice seal population parameters as well as on the scale of their exploitation. In some cases, exploitation was thought to be responsible for severe depletion of populations, such as Caspian seal, Ladoga ringed seal and others [9–11]. This was apparently not the case for ice-associated seals in Russian Far East seas where the seal harvest was managed quite effectively and did not lead to depletion of any species. Our primary aim was to document the state-regulated harvest effort that took place in the 1970s-1990s in the Sea of Okhotsk by presenting and summarizing information obtained from previously unpublished Russian sealing log books. Our main objectives were to find and resolve discrepancies between the available log books and government records; to analyze spatial and temporal variation in catch per unit effort, age group and species composition of harvest; to bring the historical social and economic context into the study; and to discuss commercial harvest impact on the ice seal populations in the Sea of Okhotsk.

## Materials and methods

We analyzed 75 sealing log books from 1972 to 1994 (excluding 1973, 1975, and 1993 where log books were not found), recorded on board 11 small wooden-hulled sealing schooners and 12 large steel-hulled, ice-reinforced hunting-fishing vessels (HFVs). The vessels carried skiffs that were used by the seal hunters to reach the sea ice and seals (S1 Fig). The log books contained the following information (in some cases partial): vessel name; year; date; daily averaged latitude and longitude of vessel position; ice concentration; ice structure; ice thickness; visibility; wind speed and direction; harvest conditions; number of skiffs; number of hunters; number of rifles; number of seals harvested per day and since the start of the harvest season (by species and age class including pups, molted pups, and adults); number of skins and pelts stored, number of carcasses and amount of meat, bones and blubber stored; number of each species wounded (i.e., struck-and-lost); name of enforcement officer on board (if any); and name of the captain.

Data from log books were manually entered and merged into a common database. Further, records were checked for typos and mathematical errors. Whenever changes or corrections were made in the data, corresponding comments were added to the appropriate record. Further cleaning of the data involved correction of outliers in geographical coordinates (mainly positions on land or in the open ocean away from vessel tracks caused by log book typos) by taking the midpoint between the vessel location the previous and the following day. In cases when the first or the last track waypoint appeared to be an outlier, the waypoint was placed on a line being a continuation of the last available straight-line portion of the track. The distance to this new point was estimated as the time elapsed since the previous waypoint, multiplied by the average rate of travel between the previous two waypoints. Only 1.2% of locations required correction. The complete database is presented as S1 Dataset.

Sea ice conditions were retrieved from the Gridded Monthly Sea Ice Extent and Concentration dataset [12] for April 1972–1994. Each year was classified into three categories depending on the observed maximum for ice coverage in this month: high ice coverage years (ice cover >70%), medium ice coverage years (ice cover from 51 to 70%), and low ice coverage years (<51%). Differences in harvest parameters (catch per unit effort as number of seals caught by one skiff in one day (CPUE), total number of vessel days, and total catch for each species) in relation to year class were tested with the Kruskal-Wallis non-parametric rank sum test [13]. We used logistic regression to investigate shifts in harvest start/end dates (response variable) in relation to ice conditions (binary predictor with 1 being a high ice year and 0 otherwise).

Data for each seal species were analyzed separately focusing on relative age groupings of catches, total harvest rates, and their interannual variations. Age structure of the catches was described following the original classification of seals in log books, including white-coated pups (newborns), molted pups, and non-pups (sub-adults and adults). We assumed the classifications made during the field observations were correct. Total harvest rates based on log book data were compared with available harvest records reported elsewhere in the literature. The percentage of seals of each species recorded as struck-and-lost seals) was estimated where the data were available. Additionally, we used previously unpublished data collected by two of the authors of the current study who participated in harvest cruises in 1977–1994 and recorded struck-and-lost seal numbers.

Variation in CPUE with ordinal date and year was summarized with Generalized Additive Models (GAMs; [14, 15]) using a Tweedie distribution fitted with the mgvc package in R [14]. We used simple factor smooth interactions which allowed a separate smooth for each level of a factor, with the same smoothing parameter for all levels. The terms in the model were fully penalized with separate penalties on each null space component. Model selection was performed based on the amount of deviance explained and REML score. Model validation was done with the gam.check function [14, 16]. Effects were considered statistically significant at α = 0.05. Model outcomes were plotted and visually inspected to draw conclusions on CPUE variation over time. Additionally, we used GAM to inspect variation in CPUE annual mean for each species separately and for all the species combined.

Data cleaning and mapping were performed in QGIS 2.12.0 –Lyon [17]. Ice data were extracted using Panoply 4.6.1 [18]. For data handling and analysis we used R 3.2.2 [19].

## Results

**Fig 1:**
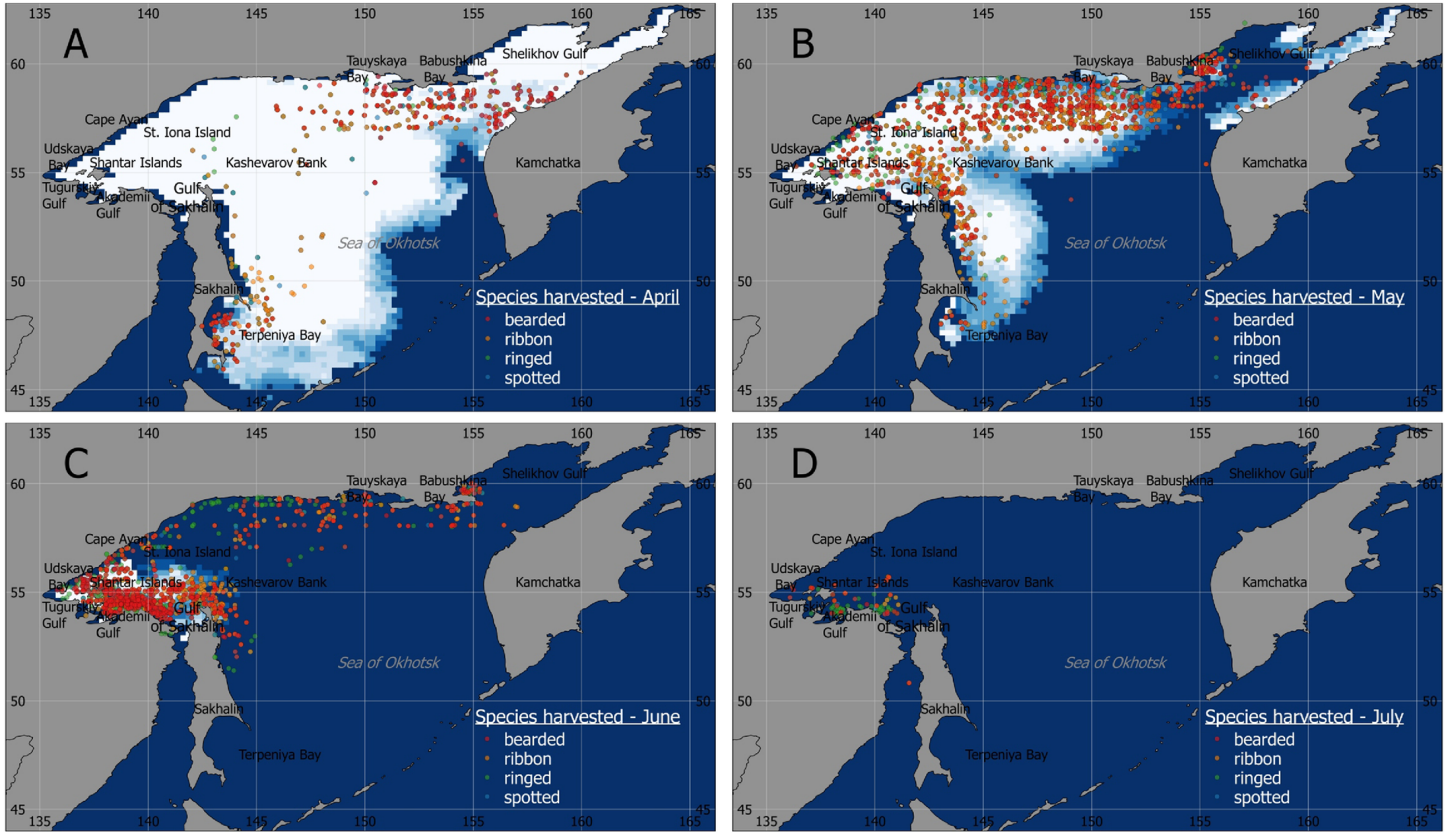
Seal harvest locations in the Sea of Okhotsk in April-July, 1972–1994 combined. Sea ice extent [12] is provided for illustrative purposes for the year of 1980 (high ice year).

The vessel-based harvest in the Sea of Okhotsk during 1972–1994 was typically conducted from early April until mid-July with the majority of seals caught in May-June (Fig 1). In 1972 and 1974, only small wooden Finnish schooners were used but starting from 1975 they were gradually replaced with larger steel ice-class Polish made vessels (HFVs) that allowed harvests in more heavily ice-covered areas. For each of the study years, log books from up to five vessels were available (Table 1). Vessels were at sea and harvesting on average for 43 days annually, ranging from 3 to 72 days. In total, 3,194 vessel-days of data were processed. Each steel HFV carried from 4 to 10 skiffs and each wooden schooner carried from 2 to 5 skiffs. Skiff brigades consisted of one hunter and two assistants working independently from other skiffs, and were therefore considered as a CPUE sampling unit.

**Table 1:** Harvest effort in 1972–1994 based on sealing log books from the Sea of Okhotsk. Entries for vessels with a researcher on board are shown in *italic*, with an official on board—underlined. Total vessel number in 1973, 1975 and 1993 was 7, 3 and 4, accordingly.

Vessel	1972	1974	1976	1977	1978	1979	1980	1981	1982	1983	1984	1985	1986	1987	1988	1989	1990	1991	1992	1994	Total vessel-days	Total Years
Akiba	13	0	0	0	0	0	0	0	0	0	0	0	0	0	0	0	0	0	0	0	**13**	**1**
Belyek	22	0	0	0	0	0	0	0	0	0	0	0	0	0	0	0	0	0	0	0	**22**	**1**
Kotik	15	0	0	0	0	0	0	0	0	0	0	0	0	0	0	0	0	0	0	0	**15**	**1**
Lahtak	47	33	0	0	0	0	0	0	0	0	0	0	0	0	0	0	0	0	0	0	**80**	**2**
Larga	31	46	0	0	0	0	0	0	0	0	0	0	0	0	0	0	0	0	0	0	**77**	**2**
Megry	39	0	0	0	0	0	0	0	0	0	0	0	0	0	0	0	0	0	0	0	**39**	**1**
Morzh	35	0	0	0	0	0	0	0	0	0	0	0	0	0	0	0	0	0	0	0	**35**	**1**
Nerpa	0	40	0	0	0	0	0	0	0	0	0	0	0	0	0	0	0	0	0	0	**40**	**1**
Olennitsa	45	0	0	0	0	0	0	0	0	0	0	0	0	0	0	0	0	0	0	0	**45**	**1**
Penza	0	25	0	0	0	0	0	0	0	0	0	0	0	0	0	0	0	0	0	0	**25**	**1**
Sivuch	28	0	0	0	0	0	0	0	0	0	0	0	0	0	0	0	0	0	0	0	**28**	**1**
Voyampolka	0	26	0	0	0	0	0	0	0	0	0	0	0	0	0	0	0	0	0	0	**26**	**1**
Zagorskiy	0	0	0	0	0	0	0	0	0	43	0	0	0	0	55	49	0	57	3	0	**207**	**5**
Zakharovo	0	0	0	0	51	0	0	0	0	0	46	0	39	47	0	0	0	53	37	0	**273**	**6**
Zalesovo	0	0	*38*	20	0	0	47	18	*66*	0	0	0	0	0	0	0	0	0	0	0	**189**	**5**
Zarechie	0	0	0	0	0	0	0	*53*	46	28	0	0	43	56	51	0	45	0	0	0	**322**	**7**
Zaslonovo	0	0	0	27	26	0	0	0	0	0	0	53	0	0	62	0	0	0	57	53	**278**	**6**
Zubarevo	0	0	0	0	0	0	29	0	0	0	0	48	0	44	0	72	0	0	0	0	**193**	**4**
Zubovo	0	0	0	0	0	0	0	54	0	0	60	0	*35*	48	0	8	0	53	28	0	**286**	**7**
Zverevo	0	0	0	0	40	45	0	0	44	52	*52*	54	0	0	60	0	0	51	0	0	**398**	**8**
Zveroboy	0	0	32	0	0	0	0	0	0	0	0	0	0	0	0	0	0	0	0	0	**32**	**1**
Zvyagino	0	0	*27*	0	0	49	0	0	52	0	0	0	51	0	0	0	0	0	0	0	**179**	**4**
Zykovo	0	0	0	0	47	*60*	0	0	58	48	49	41	*32*	0	0	0	57	0	0	0	**392**	**8**
**Total vessel-days**	**275**	**170**	**97**	**47**	**164**	**154**	**76**	**125**	**266**	**171**	**207**	**196**	**200**	**195**	**228**	**129**	**102**	**214**	**125**	**53**	**3194**	**-**
**N log books**	**9**	**5**	**3**	**2**	**4**	**3**	**2**	**3**	**5**	**4**	**4**	**4**	**5**	**4**	**4**	**3**	**2**	**4**	**4**	**1**	**-**	**-**
**Total vessels (official)**	**9**	**5**	**3**	**3**	**4**	**3**	**2**	**3**	**5**	**4**	**4**	**4**	**5**	**4**	**4**	**4**	**4**	**4**	**4**	**1**	**-**	**-**

**Fig 2:**
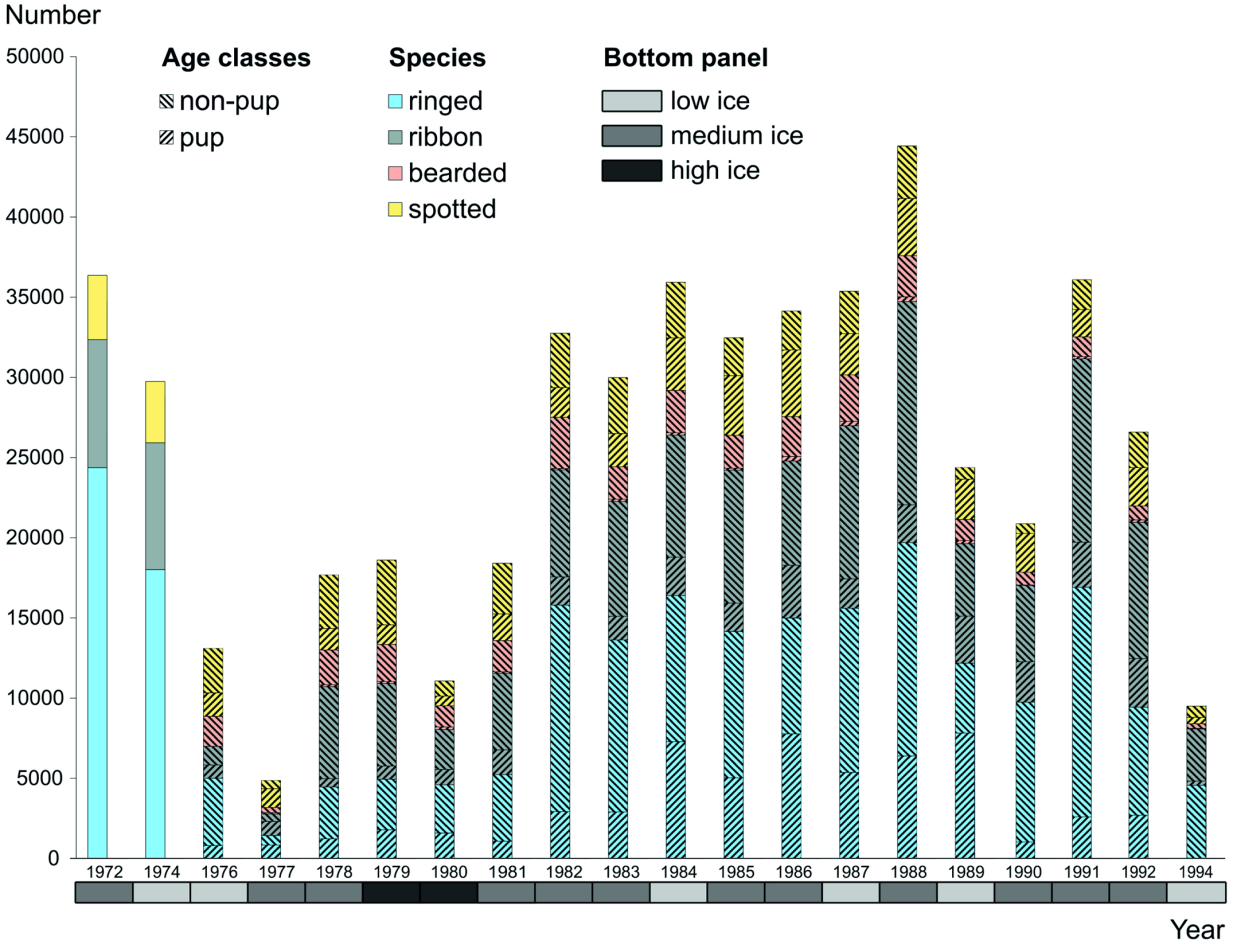
Total numbers of harvested seals by species and year in the Sea of Okhotsk based on log book data. Age class was not reported for 1972 and 1974. Bottom horizontal bar shows year category: high ice coverage years (ice cover >70%), medium ice coverage years (ice cover from 51 to 70%) and low ice coverage years (<51%).

The total numbers of seals harvested during the study period and reported in log books were: 231,879 ringed, 159,241 ribbon, 34,858 bearded, and 90,359 spotted seals (Fig 2). We compared harvest rates reported in the log books with harvest records reflected in the literature [1–7] and with the official reports of the regional division of the Ministry of Fisheries of the U.S.S.R. (Okhotskrybvod). The mean proportion of data on total harvest available from log books in relation to total vessel-based harvest reported elsewhere was 0.95 (range 0.5–1.31) for ringed seal, 1.0 (range 0.5–2.26) for ribbon, 0.91 (range 0.22–1.3) for bearded, and 0.93 (range 0.5–1.03) for spotted seal (Table 2). For some years, numbers reported in log books for a particular species slightly exceeded previously published harvest rates. For all species combined, total numbers of seals caught were within reported rates for 18 out of 20 years analyzed.

**Table 2:** Vessel-based seal harvest totals based on sealing log books and official vessel and shore-based harvest rates according to Okhotskrybvod reports from 1972–1994 in the Sea of Okhotsk. Note that these data do not include seals harvested in the west coast of Kamchatka (under jurisdiction of Kamchatrybvod). Abundance estimates are based on available population aerial survey results.

	Ringed			Ribbon			Bearded			Spotted			Total		
Year	logbook	vessel	shore	logbook	vessel	shore	logbook	vessel	shore	logbook	vessel	shore	logbook	vessel	shore
1972	24,372	24,324	2,349	7,988	7,391	0	0	0	810	4,012	4,021	20	36,372	35,736	3,179
1973	NA	25,066	4,573	NA	6,000	0	NA	0	766	NA	4,350	255	NA	35,416	5,594
1974	18,020	18,084	4,760	7,911	3,508	0	0	0	681	3,818	4,317	112	29,749	25,909	5,553
1975	NA	16,841	2,346	NA	3,500	0	NA	1,114	321	NA	3,800	137	NA	25,255	2,804
1976	5,000	5,000	1,009	2,000	2,000	0	1,862	1,859	368	4,250	4,289	115	13,112	13,148	1,492
1977	1,440	1,910	672	1,340	2,000	0	408	668	219	1,683	2,527	35	4,871	7,105	926
1978	4,458	4,500	1,084	6,262	6,000	0	2,283	2,328	396	4,694	4,694	248	17,697	17,522	1,728
1979	4,939	5,000	743	5,994	6,000	0	2,431	2,300	270	5,259	5,121	28	18,623	18,421	1,041
1980	4,593	4,593	1,305	3,451	3,451	0	1,490	1,440	176	1,540	1,540	158	11,074	11,024	1,639
1981	5,231	5,000	1,372	6,324	6,264	0	2,037	2,033	570	4,820	4,807	644	18,412	18,104	2,586
1982	15,801	15,944	1,887	8,435	10,000	0	3,288	3,224	715	5,243	5,243	369	32,767	34,411	2,971
1983	13,630	13,630	2,417	8,642	8,639	0	2,167	2,168	618	5,562	5,563	869	30,001	30,000	3,904
1984	16,405	16,367	1,568	10,000	10,000	0	2,791	2,845	605	6,744	6,700	575	35,940	35,912	2,748
1985	14,160	14,259	2,989	10,060	10,000	0	2,175	2,150	809	6,086	6,024	822	32,481	32,433	4,620
1986	14,996	15,901	2,925	9,802	10,000	0	2,763	3,089	477	6,598	7,046	784	34,159	36,036	4,186
1987	15,599	15,599	4,590	11,393	11,418	0	3,173	3,018	694	5,222	5,198	731	35,387	35,233	6,015
1988	19,693	15,000	4,281	15,050	14,928	0	2,866	2,211	775	6,834	6,847	861	44,443	38,986	5,917
1989	12,190	23,385	3,923	7,450	14,900	75	1,512	2,931	778	3,250	6,500	859	24,402	47,716	5,635
1990	10,000	19,860	2,512	7,450	14,695	11	865	3,994	432	3,388	6,263	321	21,703	44,812	3,276
1991	17,354	17,343	2,195	14,638	14,626	18	1,392	1,954	415	5,659	5,659	352	39,043	39,582	2,980
1992	9,440	9,406	1,677	11,532	11,543	150	1,025	1,019	356	4,603	4,553	364	26,600	26,521	2,547
1993	NA	12,205	1,441	NA	13,447	90	NA	1,650	394	NA	4,169	334	NA	31,471	2,259
1994	4,558	4,578	354	3,519	3,519	23	330	333	101	1,094	1,094	360	9,501	9,524	838
Abundance estimate	500,000 [9]			150,000 (1970s); 400,000 (late 1980s) [9]			70,000–195,000 [9]			120,000–200,000 [9]			-		

According to population estimates from aerial surveys in the second half of the 20th century (Table 2) and the harvest rates reported earlier [1], between the 1960s and 1990s from 1.1 to 5.7% of ringed seals, 1.2 to 4.3% of ribbon seals, 0.1 to 6.2% of bearded seals, and 2.5 to 10.9% of spotted seals were taken annually.

In most years (except 1971, 1977, 1978 and 1979), the harvest was predominantly represented by ringed seals (mean = 0.43, range 0.25–0.67), followed by ribbon (mean = 0.31, range 0.15–0.43), spotted (mean = 0.19, range 0.11–0.35), and then bearded seals (mean = 0.07, range 0.03–0.14).

Out of 75 harvest cruises over the 22-year period, enforcement officers were present on 49 of them (see Table 1). Their main responsibility was to control the harvest process, maintain compliance with regulations, and data entry in the log books. Nevertheless, the enforcement officers did not necessarily record information on the numbers of seals struck-and-lost: log books from only 31 out of 75 cruises contained these data. Therefore, we can determine only the minimum struck-and-lost rate because the actual losses were higher. Minimum harvest losses varied from 0.1 to 21.9% of total ringed seals caught per vessel per season, from 0.1 to 18.3% for ribbon seals, from 0.3 to 31.4% for bearded seals, and from 0.1 to 18.3% for spotted seals. From 1972 to 1994, losses of seals during harvest reported in logbooks were 5,557 (2.4% of total harvest) ringed, 3,240 (2.3%) ribbon, 1,923 (5.1%) bearded, and 2,569 (3.1%) spotted seals. According to the enforcement officers present onboard, and Okhotskrybvod reports, seal harvest losses were 30–35% of the catch for ringed, bearded and spotted seals and 15–20% for ribbon seal (A. Somov, pers. comm.), hence the overall human-induced mortality was at least 15% higher than the harvest for ribbon seal and 30% higher for the other three species.

Starting in 1975, harvested seals were divided into age classes. The proportion of pups, both white-coated and molted, taken in catches during 1975–1994 remained stable for all species throughout the study period (Fig 2) with very low numbers of pups harvested before molting. Although, the timing of pup harvest somewhat differed between years, molted pups, in most cases, were hunted during the entire harvest season (S2 Fig). Based on our data, 59,054 molted ringed seal pups, 31,529 ribbon, 2,462 bearded, and 37,419 spotted seal pups were harvested. For spotted seal, molted pups composed over 52% of all catches. Ringed seal white-coated pups (260 for all years combined) were reported in harvests from April 13 to May 10 with a single white-coated pup caught on June 20 in 1978. Ribbon seal white-coated pup catches (646) were reported from April 11 to May 11 with single pups caught later in the season on May 29 in 1977 and June 19 in 1979. Spotted seal pup catches (802) were reported from April 8 to May 15 with single pups caught on June 6 in 1980, June 7 in 1977 and June 18 in 1981. Twenty-five bearded seal pups were reported to be caught in lanugo coat during 1979 (from June 10 to June 29), on June 20, 1986, and April 12, 1988. Overall, the small sample sizes of pups in their lanugo pelage did not support reliable conclusions on pupping and molting timings.

CPUE was estimated for all the data points where the number of skiffs working at sea was recorded in log books (n = 3,123) as the number of seals of each species caught by one hunting brigade in one day. CPUE for ringed seals was the highest (mean = 11.55, max = 200), followed by ribbon (mean = 6.79, max = 86), spotted (mean = 4.17, max = 65), and finally bearded seals (mean = 1.82, max = 33). For all species combined, each skiff on average harvested a mean of 24.31 seals per day (interquartile range = 11–33.33, max = 200).

**Fig 3:**
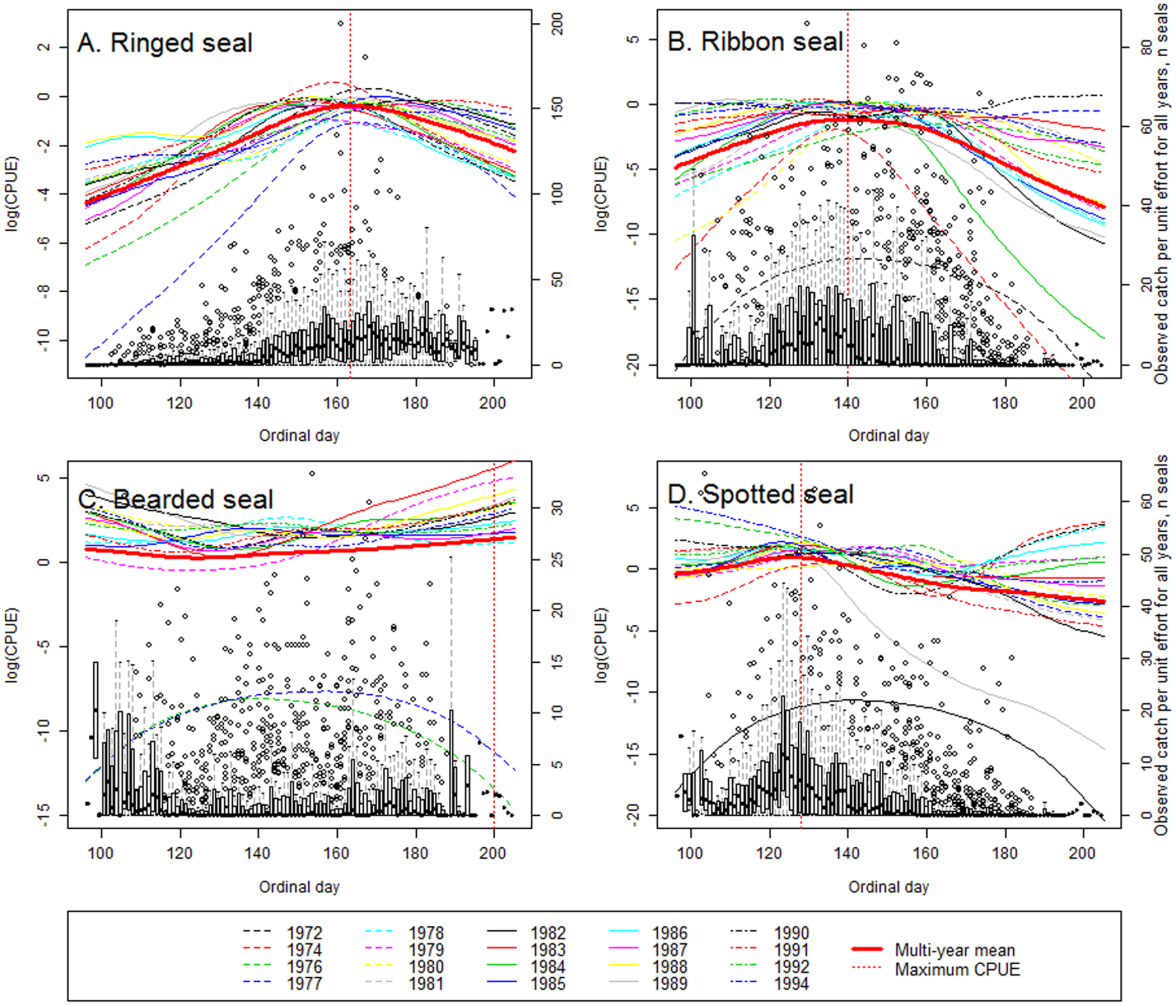
Observed catch per unit effort for 1972–1994 combined (right Y-axis) plotted against ordinal day. The boxes show the number and range of 50% of observations in each group; bold horizontal lines in boxes indicate median number of seals caught, dots indicate outliers in the data. The left Y-axis is for the GAM smooths for each year and multi-year mean. Note that both Y-axis scales vary with species.

Ordinal day and year had significant (*p*<0.05) effects in the GAM regressions on CPUE for all four species. CPUE for ringed, ribbon, and spotted seals appeared to have a similar pattern in relation to day of the year (Fig 3A, 3B and 3D) with one distinct spike in catches that occurred first for spotted seals (on average, on May 7), followed by ribbon seals (May 20), and then ringed seals (June 11). Visual inspection of plots suggested that CPUE for ringed and ribbon seals was lower, in general, for 1972–1981 compared with later years. After mid-season catches of ribbon seal appeared to decline rather rapidly. CPUE of spotted seals demonstrated no major visible difference between the years: lines clustered together with gradual decline towards the end of the season. The year 1982 was an exception for the spotted seal harvest with very low catches. Catches of bearded seals showed a less pronounced temporal trend with gradual increase toward the end of the season in the majority of years (Fig 3C). The years 1972, 1974, 1976 and 1977 stood out from the remainder, with much lower CPUEs in all the species; bearded seals were not reported in catches in 1972–1974 as the quotas existed only for the shore-based harvest at that time.

**Fig 4:**
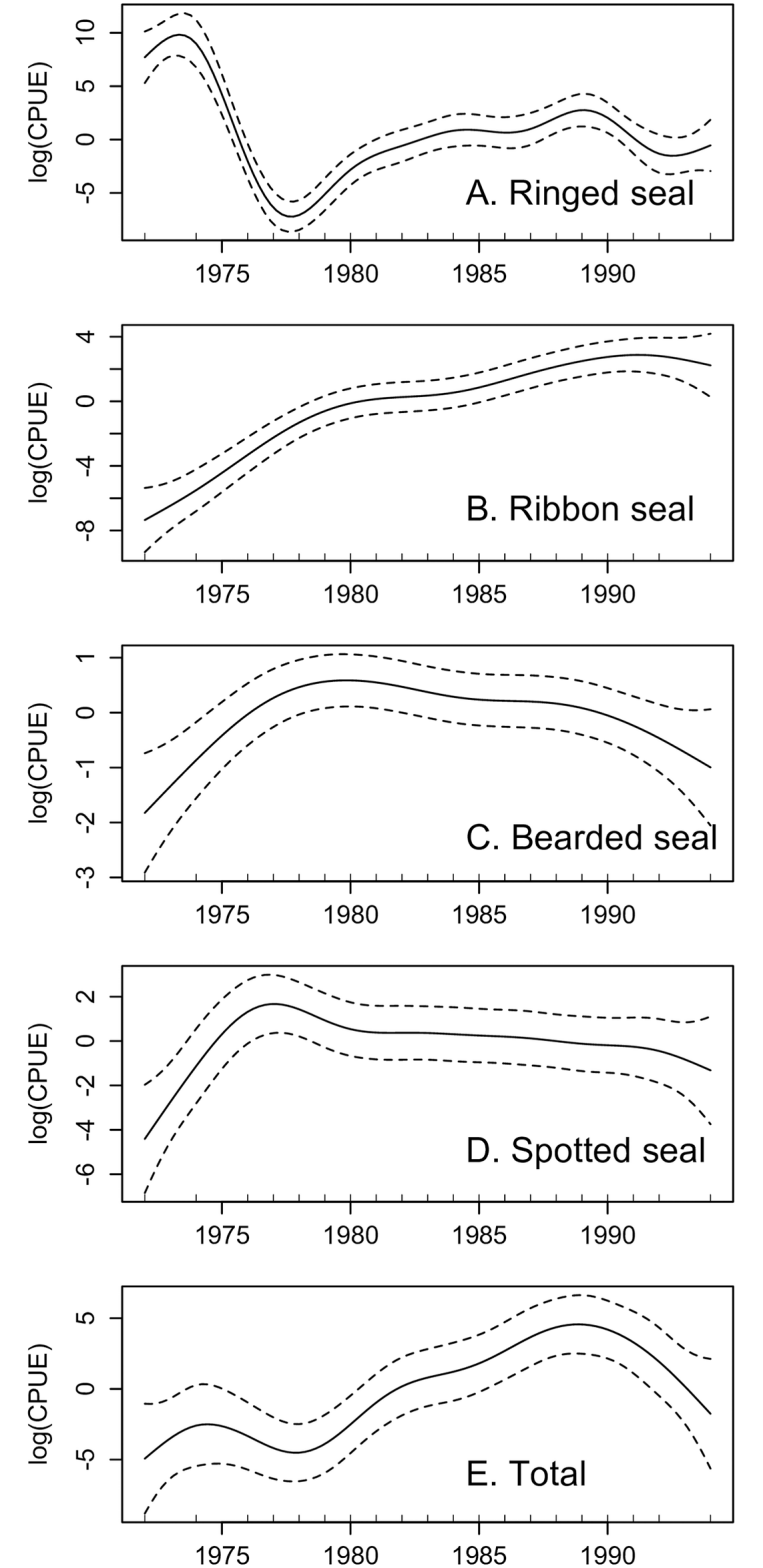
GAM fit and 95% CI for mean observed catch per unit effort, 1972–1994. Note that the Y-axis scales vary with species.

The trends in annual mean CPUE were notably non-linear in all four species, with the possible exception of ribbon seals (Fig 4). For the ringed seal, the period of regulated harvest began with high levels of CPUE in 1972–1974 followed by an abrupt decline in this parameter in 1975–1977, and then a gradual increase and a plateau in daily catches during the rest of the study period (Fig 4A). Ribbon seal CPUE underwent a steady, close to linear increase (Fig 4B). Model outcomes for bearded (Fig 4C) and spotted (Fig 4D) seals were structurally similar to one another: CPUE increased in the first half of 1970s, hit a plateau around 1978, and remained stable with a slight decrease towards the end of the period. Finally, the temporal trend in mean annual CPUE for all seals combined mostly reflected that of the ringed seal which was the dominant species in catches and hence harvest success in term of this species was reflected in the overall pattern (Fig 4E).

In 1972–1994 (excluding 1973, 1975 and 1993) there were 2 years with high ice coverage (1979 and 1980), 6 years with low ice coverage and 12 years with medium ice coverage (see Fig 2, bottom bar). The sealing started as early as the first half of April even in high ice coverage years and typically lasted until late June or early July, whereas the ice melted completely, on average, by mid-July. A logistic regression model showed a marginally significant effect (ANOVA Chi-squared test, *p* = 0.074) of year class on harvest start date suggesting that, perhaps, on low ice-coverage years (ice coverage <51%) the harvest tended to start later than on medium and high ice-coverage years. The effect of the ice-coverage on harvest end date was not significant. There were no significant differences revealed in CPUE, number of vessel-days and total catches for each species per year in relation to ice-coverage class (Kruskal-Wallis rank sum test, *p*>0.1).

Spatial patterns of harvest indicated that for all four species it typically began in Shelikhov Gulf in the northeast Sea of Okhotsk and Terpeniya Bay off the southeast coast of Sakhalin Island. As the season progressed and sea ice cover was breaking up and retreating from northeast to southwest, the sealing vessels followed the retreating ice edge where the largest concentrations of seals could be found (Fig 1 and Figs A-P in S1 File).

In April, ringed seal harvest in terms of CPUE was most successful in Terpeniya Bay and in the coastal waters on the northern Sea of Okhotsk from the easternmost end of Tauyskaya Bay to Babushkina Bay (Fig A in S1 File). In May, catches were rather evenly distributed across all harvest zones in the Sea of Okhotsk with more ringed seal being caught in shorefast ice areas (Fig B in S1 File). Later in summer months, distinct harvest “hot spots” were found in the waters adjacent to Shantar Islands; (the so called Shantar Sea)–in Udskaya Bay, Tugurskiy Gulf and Akademii Gulf, where the remaining ice fields were located (Fig C,D in S1 File).

Ribbon seal harvest began in April mainly in Terpeniya Bay and farther north along the eastern coast of Sakhalin (Fig E in S1 File). Noticeably, this species was caught exclusively in the ice edge zone on floating ice. Similar to ringed seal catches, ribbon seal catches in May were distributed more evenly across the sea, but the numbers of ribbon seals far away from shore were higher with “hot spots” observed northeast of Sakhalin Island and south of Tauyskaya Bay (Fig F in S1 File). The same concentrations of seals in northern Sakhalin waters were apparently exploited in June (Fig G in S1 File), whereas much smaller catch rates were reported for July in Udskaya Bay, part of the Shantar Sea, and Sakhalin west coast (Fig H in S1 File).

The bearded seal is the only one of the four species where a high harvest occurred along the western Kamchatka coast and in Shelikhov Gulf in mid-spring. (Fig I in S1 File). Later in the season catches were reported along the entire western shore of the Sea of Okhotsk with a minor gap around 58°N (Fig J in S1 File). In June and July, bearded seal distribution was similar to those of ringed and ribbon seals, but they were found much deeper in bays and gulfs of the Shantar Sea than the other species. (Fig K,L in S1 File).

Spotted seals were taken in several different locations in April, including Terpeniya Bay and areas south of Tauyskaya Bay (Fig M in S1 File). Vessel locations with the highest CPUE in May were mainly concentrated between 145° and 155°E, south of the coastline (Fig N in S1 File), then became closer to the shore, and formed a typical “hot spot” in the Shantar Sea later in June (Fig O in S1 File). Spotted seal catches in July were very low compared with other species (Fig P in S1 File).

## Discussion

### Harvest history

In Russia, marine mammal hunting was a major component of traditional subsistence activities and was common in many coastal indigenous peoples’ communities including those located in the Russian Far East along the Sea of Okhotsk. A number of ethnic groups relied on marine mammals as a major source of food and essential materials. For instance, in the beginning of the 20th century, 63% of Koryak households on the Kamchatka peninsula were engaged in seal or whale hunting [20]. Nivkhi on Sakhalin and in the Amur River estuary were known to hunt sea mammals, especially seals and sea lions, using floating harpoons and clubs from boats or from the shore. Negidal, Evens, Nivkhi and some other indigenous peoples have hunted marine mammals off the coast of the Sea of Okhotsk since the 18th century using bidarrahs (a multi-seat baidarka), kayaks and other types of boats [21].

During an intensive period of growth of settlements in the early to mid-20th century, commercial sealing started as coastal seal hunting and then became well developed with construction of specialized sealing vessels in the late 1950s and early 1960s [7]. The commercial seal harvest established in the Russian Far East in 1932 was unregulated until 1968 for all the species hunted. Until the late 1960s, harvests were mainly for blubber and skin and took place predominantly on molting grounds [22]. Due to both a lack of harvest limits and non-selectivity of catches, harvest data were extensively used to draw conclusions on a populations’ status, as catches were presumed to reflect seal abundances [9]. Scarce information available on seal catches before the 1950s suggests that aboriginal seal hunting did not exceed 25,000–35,000 individuals for all species combined per year in the northern part of the Sea of Okhotsk [6]. According to Fedoseev’s estimates [9], in the years of intensive unregulated sealing, 1955 to 1968, the average annual catch was about 78,500 ringed seals, 13,000 ribbon seals, 10,100 bearded seals, and 4,600 spotted seals.

By 1963, high harvest rates led to depletion of seal stocks and the catches started declining [6]. In 1968, hunting limits were set on vessel- and shore-based harvests, wherein hunting of ringed and spotted seals was permitted only from May to July [23]. Ribbon seal harvests were restricted to 7,000 per year due to stock decline. At the same time, Fedoseev [22] pointed out that the harvest limits introduced initially were biased as they did not take into account unbalanced exploitation of the regional structure in seal populations; dense reproductive concentrations of seals were under major pressure and continued rapidly toward depletion.

We interpret a trough in total harvest rates appearing in the second half of the 1970s (Fig 2) to be a consequence of a science-based decision made by state authorities to reduce total allowable catch rates for all seal species in the Sea of Okhotsk. The purpose of this was to allow the populations to recover from a period of uncontrolled exploitation followed by several years of high harvest quotas. The quotas were substantially increased again in 1982.

In 1968–1975 the Soviet government issued a series of decrees ordering the development of fur-oriented sealing. This caused harvests to become highly unsustainable: small schooners were unable to keep the meat on board so they discarded it, and because the main target of sealers shifted towards molted individuals, a major portion of seals killed were thrown overboard due to insufficient fur quality. Hunting of bearded seals was virtually stopped due to the low value of its pelt [5, 6, 22].

In the early 1990s, after the collapse of the Soviet Union, the state ceased subsidizing the sealing industry and the seal harvest collapsed. Since 1995, sealing has been conducted in the Sea of Okhotsk only by enterprises representing the interests of indigenous peoples and their total harvest did not exceed 1–4% [7] of the quota of approximately 50,000 seals for all species combined, allocated to the Magadan Region in 1995–2005. Starting in 2006, Total allowable seal catches were reduced drastically to 7,800 in 2008 and 2,100–2,200 in 2009–2012. After 2012, ice-associated seal species were moved from "Total allowable catches" system for the Sea of Okhotsk to "Possible catches" which is a less strict system used for less commercially valuable species with low harvest interest [24–44].

The shore-based harvest pressure on seals has always been lower compared with vessel-based harvest pressure. We found records on shore harvest rates in Okhotskrybvod reports from the study period (see Table 2). Also, Trukhin [1] summarized those rates for a period from 1947 to 2004. For the years in which the data on both types of harvest were available, the mean contribution of shore harvest to the overall seal take in the Sea of Okhotsk was 0.26 (range 0.035–0.893) for ringed seals, 0.28 (range 0.093–0.721) for bearded seals, and 0.11 (range 0.004–0.485) for spotted seals. Ribbon seals were not harvested from shore (except low numbers reported in catches in 1989–1994 and 2004). To the best of our knowledge during the 20th century, 353,003 ringed, 29,329 bearded, and 24,697 spotted seals were harvested from shore [1].

### Reliability of log book data

We consider log book data analyzed to be a representative sample of the total harvest effort for the study period: based on official records on total number of vessels working in 1972–1994 provided by the Russian Federal Research Institute of Fisheries and Oceanography (VNIRO) and reported by Okhotskrybvod, we possess data from approximately 94% of the sealing cruises. Nevertheless, we encountered certain discrepancies between catch rates for particular species recorded as the harvest progressed, and harvest rates reported later in the literature [1–7]: since the sources of information were often not reported in the scientific publications, we believe that the values of harvest rates might have been partially estimated based on incomplete information available to the authors at that time. While analyzing raw log book records, we also noted that certain numbers of seals initially recorded as taken for their pelts were later moved to the record field reflecting that they were taken for their meat, or vice versa. Some low numbers of mathematical or typing errors were found in the logs. Mapped seal catch locations (Figs A-P in S1 File) based on log book data sometimes appeared to follow straight lines going from north to south or from east to west. This is an artifact of the specifics of the geographical coordinate format provided in the sealing log books, where latitude and longitude were rounded to two decimal places due to the absence of more precise geolocation systems.

Harvest and fishing vessels working at sea were obliged to accommodate an enforcement officer on board if one was provided. Based on log book records, officers were only present on some cruises and could work only on one of the skiffs, whereas most vessels had several skiffs. This officer, generally either a state official or biologist from a research institution, was empowered to enforce seal harvest regulations according to sealing areas and quotas as well as proper reporting. State officials could be involved in harvests directly as hunters (and therefore receive additional income). In these cases, we assumed that the reliability of the inspection results could have been compromised due to extra work duties performed and potential conflicts of interest.

We did not discern any relationship between the presence of an enforcement officer on a vessel and the accuracy of the records, or the consistency in reporting of harvest losses. For instance, even though vessels had an officer on board, daily harvest rates for all species were apparently rounded (100, 150, 200, etc.) on some cruises during 1976. We also found several instances when the same person was listed as an enforcement officer on two different vessels simultaneously working at sea. Although we believe that the discrepancies described above do not diminish the overall value of the data preserved in the log books, they should be borne in mind when interpreting our results and drawing specific conclusions.

### Temporal variation in catches

We hypothesized that harvest start dates differed somewhat depending on ice coverage in April. Although we expected harvest to start later in years with heavier ice conditions, the model outcome showed the opposite trend. According to the recollections of the authors of the present study who participated in the harvest cruises, use of HFVs removed the limitations associated with ice conditions and the vessels began the harvest depending on organizational and technical aspects, mainly vessel maintenance, expedition supply, and crew recruiting. At the same time, the lack of a significant effect of ice conditions on catch rates implies that within observed ranges of ice coverage in 1972–1994 sealers always managed to take their quotas, apparently due to abundant seal populations.

In the Sea of Okhotsk we did not find any pronounced trends in the frequency of high ice years or low ice years, neither during the study period nor in the following years. Based on our classification, the proportion of high ice years slightly decreased from 0.14 to 0.09, in low ice years from 0.41 to 0.32, and in medium ice years increased from 0.45 to 0.59 when comparing data from 1972–1994 with data from 1995–2013. Finer scale data are needed to demonstrate a possible effect of ice conditions on seal population abundances and harvest success.

The beginning of sealing season in 1972–1994 typically coincided with peak pupping season for ringed seals, and early pupping season for ribbon, bearded and spotted seals in the Sea of Okhotsk [45–48]. The presence of white-coated pups reported in catches in April to mid-May for ringed, spotted and ribbon seals corresponds with the general timing of molt in pups of these species [45–48]. Bearded seal pups in their natal coats, which is brown, are unlikely to be found in the catches in high numbers as many of the pups complete at least part of their molt *in utero* [49]. Single records of natal-coated bearded seal pups in catches from June-July might be a result of delayed parturition of individual females (possibly young) or data entry errors.

Ringed seal CPUE increased gradually from the beginning of harvest to reach its maximum level only in mid-June, later than the CPUE of the other three seal species (see Fig 3A). Ringed seals occur primarily in shorefast ice regions not easily accessible to sealing vessels in April; this explains the larger CPUEs later in the season when ice started melting. Low catch values early in the 1972 and 1974 seasons may be due to the use of smaller wooden boats incapable of penetrating the compact ice zones. Another reason for low catches might be that at least some pups are born in under-snow lairs (especially for ringed seals breeding on shore-fast as opposed to the drifting ice in the Sea of Okhotsk; [9, 50, 51]). Those seals would become available for sealers only after the snow melts. After reaching its maximum rate, ringed seal CPUE remained more or less stable until the end of the harvest season.

Ribbon seal CPUE (see Fig 3B) followed a more or less symmetric curve, peaking in late May when these seals transition from nursing to molting season. Ribbon seals spend comparably more time on floating ice in the ice-edge zone [45] than other ice-associated species. They were a conspicuous and important harvest species throughout the season. The gradual decline in observed catches in July could be explained by a highly-dispersed distribution that did not provide harvest concentrations of sufficient size.

Low bearded seal CPUE, in comparison to the other species, could be related to the ability of bearded seal females and pups to escape into the water during the nursing season, and a generally lower interest in this particular species from hunters. Bearded seal catches were slowly increasing, presumably due to the beginning of the molt, from late May to early June and continued to do so until the end of the harvest season (see Fig 3C).

Spotted seal harvest seemed to have been more pup-oriented compared with other species as it primarily took place on pupping grounds. Outside the breeding season, adult spotted seals on ice were characterized [52] as having low disturbance tolerance and hence being difficult to approach. Also, the hunters were encouraged to start harvesting from areas with high spotted seal concentrations in order to fulfill their catch quota. This explains an early spike in spotted seal catches and then a rapid and constant decline in numbers of this species harvested (see Fig 3D).

Changes in CPUE annual mean from year to year may be linked to organizational aspects of harvest operations, changes in harvest techniques, and staffing problems. Starting in 1975, new ice- reinforced vessels replaced the schooners and this change required additional staffing and training of new crews. The Korsakovskaya oceanic fishery government enterprise became responsible for vessel and crew preparation for the harvest season and the Okhotskrybvod later reported a highly unsatisfactory level of performance: there was not enough warm clothes, food supplies, and safety and shooting training provided to the newly employed vessel crews. Lack of sufficient hunting experience in a number of hunting brigades resulted in a two- to three-fold decline in CPUE (most pronounced in ringed seal harvests, which composed the major portion of catches), substantial increase in numbers of wounded seals and even led to an accidental injury of one of the hunters in 1977. The total time spent on-effort at sea also decreased due to testing and essential maintenance of new skiffs as well as crew at-sea training which took up to two weeks. The technical and staffing problems were gradually overcome after 1978 which resulted in increasing trends in catches (see Fig 4). A steady increase in ribbon seal CPUE could be related to the population recovery after an earlier period of uncontrolled exploitation, whereas increase of bearded seal take was mostly driven by abandonment of the fur-focused harvest which revived interest in this species. The CPUE of spotted seal increased shortly after the introduction of HFVs and remained stable until the end of the commercial harvest.

Declining CPUE values in exploited populations might be a good indicator of negative changes in population size. Visual inspection of GAM outcomes did not provide clear evidence of the decline of this parameter over the study period. Temporary troughs in CPUEs are shown to be largely associated with harvest management.

### Spatial variation of catches and seal distribution

Spatial variation in catches was largely associated with vessel initial positions and harvest effort allocation. However, vessel harvest locations revealed significant differences in the distribution of catches of different seal species, which supported inferences about actual seal distributions on spring ice.

The ringed seal stands apart from other ice-breeding species due to its ability to maintain a system of water access and breathing holes in thick fast ice throughout the ice-covered period (e.g. [53]). This allows ringed seals’ breeding range to extend farther from the ice edge zones to avoid competition with other species for resources, and provides more access to ice features (hummocks and ridges; [9]) and dense snow cover as a shelter for their new born pups (e.g. [54]). In the Sea of Okhotsk, ringed seals have been found occasionally breeding on drifting ice where they do not always have the opportunity to construct birth lairs [51].

Based on aerial survey maps published in various years, ringed seals are fairly evenly distributed throughout their range in the Sea of Okhotsk [55–59]. However, the seal herds were not exploited equally by the sealers, which could lead to unbalanced exploitation of the most productive areas [9]. In April, for instance, large densities of ringed seals were reported in the Shantar Sea, but ice conditions early in the season prevented sealing vessels from working in this area.

Harvest data for 1972–1994 demonstrate that the main concentrations of ringed seals in April-May were closely associated with the near-shore fast-ice zone. A well-defined band of high seal density occurred along the coast from Shelikhov Gulf in the North to the northern Shantar Sea waters and the northern part of Sakhalin Island in the South. Fedoseev [56] described the breeding herd distribution for ringed seals and suggested that the groups of breeding seals south of the line from Ayan to St. Iona Island tended to concentrate on the so-called Shantarskiy ice massif. The other groups of breeding herds, located northward of 56°N, formed molting aggregations in the Okhotsk-Tauyskiy region. Breeding ringed seals were observed in Shelikhov Gulf, near the eastern Sakhalin and in the northern part of Terpeniya Bay [9]. In May, seals moved to molting grounds closer to the shore: north from Terpeniya Bay and north-west, west and south from the central Okhotsk Sea [60]. Based on our data (Fig B,C in S1 File), ringed seal molting herds, similar to other species, were associated with the remaining ice fields in late spring—early summer and were exploited exclusively in the Shantar Sea later in July (Fig D in S1 File).

The spatial structure of the ribbon seal harvest corresponded well with this species’ distribution in the whelping and molting seasons. The ribbon seal is a typically pelagic species whose habitat is associated with zones of the continental slope. It is incapable of maintaining breathing holes in the ice and occurs primarily in the areas where vertical circulation of water masses leads to formation of large ice floes, which makes these areas rich in fish. According to Fedoseev [9], in the Sea of Okhotsk, ribbon seals were observed more often on ice floes located in the deep-water zone off the shelf adjacent to Terpeniya Bay. Fedoseev et al. [57–59] survey maps showed ribbon seal aggregations in April being a considerable distance from shore, and also somewhat interior to the ice, away from the edge. In years with low ice concentration, ribbon seal herds could be found in the ice-edge zone beginning early in the season [56, 61], but in high ice concentration years, high densities of ribbon seals in April were only observed off the East coast of Sakhalin [62]. Comparing harvest distribution maps for April-early May (Fig E,F in S1 File) with the results of aerial surveys carried out in peak reproductive season [56–59] there were similarities in locations of high CPUE values and concentrations of ribbon seals. In both cases there were hot spots south of Terpeniya Bay in April, shifting gradually to the waters along the northeastern coast of Sakhalin Island later in the season if the ice conditions were initially severe or occurring there already in April if the ice edge was closer to the shoreline. Those two areas of high ribbon seal densities are isolated from one another by a vast zone of compact ice fields [9], which is not a preferred habitat for the species.

A dense concentration of ribbon seals was typically located north of 58°N and was considered as a separate breeding herd isolated presumably by bathygenic inflow of the warm Pacific waters passing through the Kashevarov Bank towards St. Iona Island [9]. Based on sealing log book data this herd was exploited only in May with occasional individuals taken in April. The distribution of molting ribbon seals in May reported by Fedoseev [56] coincided with the retreating ice edge location: seals were concentrated around the Shantar Sea and the north-western parts of the Sea of Okhotsk (Fig F in S1 File).

The bearded seal is a typical benthic and epibenthic feeder that prefers shallow, continental shelf regions. In wintertime it breeds on grey, grey-white, and white ice with a thickness of typically less than 60 cm [9] that prevails in the regions adjoining the fast ice polynyas and occurs very often over the continental shelf. In the Sea of Okhotsk, bearded seals prefer to breed closer to the shore and the distribution of breeding herds is rather stable from year to year, though in some places small patches of breeding seals are scattered chaotically throughout the shelf zone [9]. Bearded seals are not dependent on stable sea ice since bearded seal pups undergo prenatal molt and are capable of entering the water soon after birth [49]. Bearded seals in the study area breed later in the season than other seal species with peak pupping in late-April to mid-May [3, 63, 64].

Compared to ringed seals, bearded seals are less active in making and maintaining breathing holes in ice and require presence of water leads and polynyas. Thus, their breeding habitat in the Sea of Okhotsk is mostly located in the drifting ice zones in Shelikhov Gulf, Tauyskaya Bay and off eastern Sakhalin [56]. Their breeding habits correspond with catch distributions in April known from sealing log books (Fig I in S1 File). The tendency of bearded seal breeding and molting herds to associate with water openings likely leads to a patchy pattern of seal distribution throughout the sea, which is illustrated by aerial survey maps [56] and is supported by harvest distributions (Fig J in S1 File).

Bearded seals in their molting season remain patchily distributed [58], but the overall extent of the species coincides with the residual ice area. Maximal catches of the species in June and July occurred in the Shantar Sea coastal waters (Fig K,L in S1 File).

Spotted seal breeding areas are very similar to the ones occupied by ribbon seals. Fedoseev [9] suspected some degree of interspecific competition for available breeding habitats and fish resources. He also described spotted seal breeding herds as occupying the northern-northwestern part of the sea. This is under the influence of warm Pacific water masses penetrating through a trench along western Kamchatka, causing broken forms of ice to prevail [9]. Our data suggest the similar distribution of major reproductive concentrations of this species in April, and also confirms the presence of large numbers of spotted seals in Terpeniya Bay (Fig M in S1 File).

There are two major molting concentrations of spotted seals [56], but mainly the northern one was exploited during the harvest in May (Fig N in S1 File), which might be explained by later weaning of spotted seal pups in the northern parts of the Sea of Okhotsk [46], and hence more successful harvests in those areas due to higher disturbance tolerance in breeding females.

## Conclusions

Commercial seal harvests in the Sea of Okhotsk not only had a significant impact on the marine ecosystem as a source of mortality for ice-associated seals inhabiting the area, but also provided a large amount of data on species biology, demography, and seasonal distribution. The distributions and timing of the harvests corresponded well with the prevailing understanding of the different species’ natural histories, and therefore our harvest data should provide an enhanced basis for future assessment of impacts from the reduction of sea ice anticipated in the Sea of Okhotsk as the Arctic climate continues to warm.

Original records contained in log books enhanced our understanding of the seal harvest and the populations that were exploited. Comparison of harvest logs with relevant information published elsewhere allowed cross-checking to identify and resolve discrepancies. The edited data set will support a planned quantitative analysis of relationships between biological patterns in ice-associated seal populations, harvest patterns, and environmental variability.

Despite substantial struck-and-lost rates and high numbers of seals harvested throughout the study period, timely introduction of state regulations and efficient harvest management apparently prevented severe depletion of ice-associated seal populations in the Sea of Okhotsk.

## Supporting information

S1 Fig Types of harvest vessels used in the Sea of Okhotsk in 1972–1994. (PDF)

S2 Fig Seal harvest timing. (PDF)

S1 Dataset Sealing log books from the Sea of Okhotsk, 1972–1994. (XLSX)

S1 File Seal harvest distribution maps by month and species in the Sea of Okhotsk, 1972–1994. (PDF)
