# Supplementary material for: The commercial harvest of ice-associated seals in the Sea of Okhotsk, 1972-1994
Source: PLoS One. 2017 Aug 10;12(8):e0182725. doi: 10.1371/journal.pone.0182725 (PMC5552157; doi:10.1371/journal.pone.0182725)
Supplement: S1 Fig — (PDF) [file pone.0182725.s001.pdf]

S1 Figure: Types of harvest vessels used in the Sea of Okhotsk in 1972 -1994

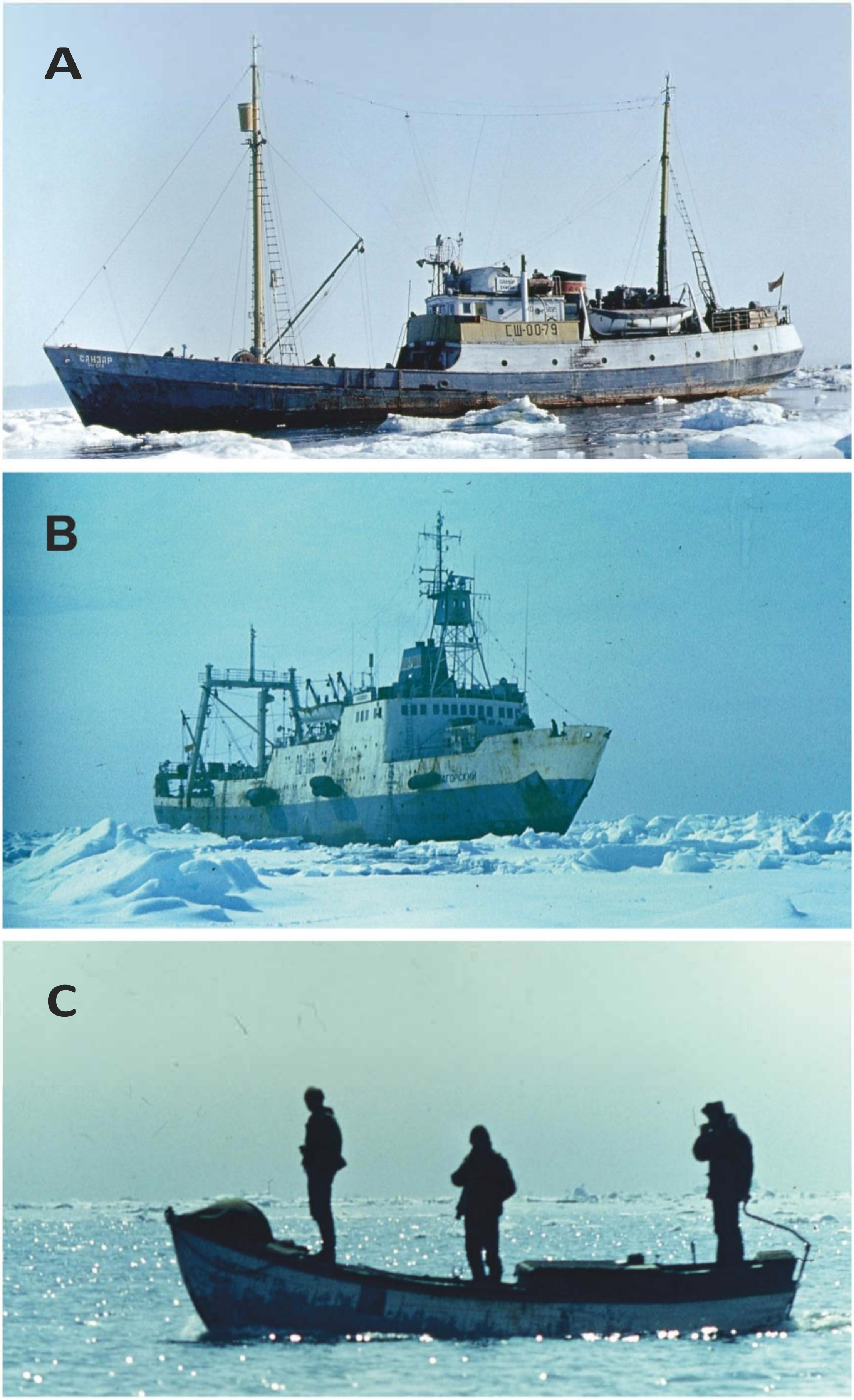

.

A - Wooden-hulled sealing schooner
B - Steel-hulled, ice-reinforced hunting-fishing vessel (HFV)
C - Skiff
(pictures from Y.A. Bukhtiyarov's archive)
