# Supplementary material for: The commercial harvest of ice-associated seals in the Sea of Okhotsk, 1972-1994
Source: PLoS One. 2017 Aug 10;12(8):e0182725. doi: 10.1371/journal.pone.0182725 (PMC5552157; doi:10.1371/journal.pone.0182725)
Supplement: S2 Fig — (PDF) [file pone.0182725.s002.pdf]

S2 Figure. Seal harvest timing.

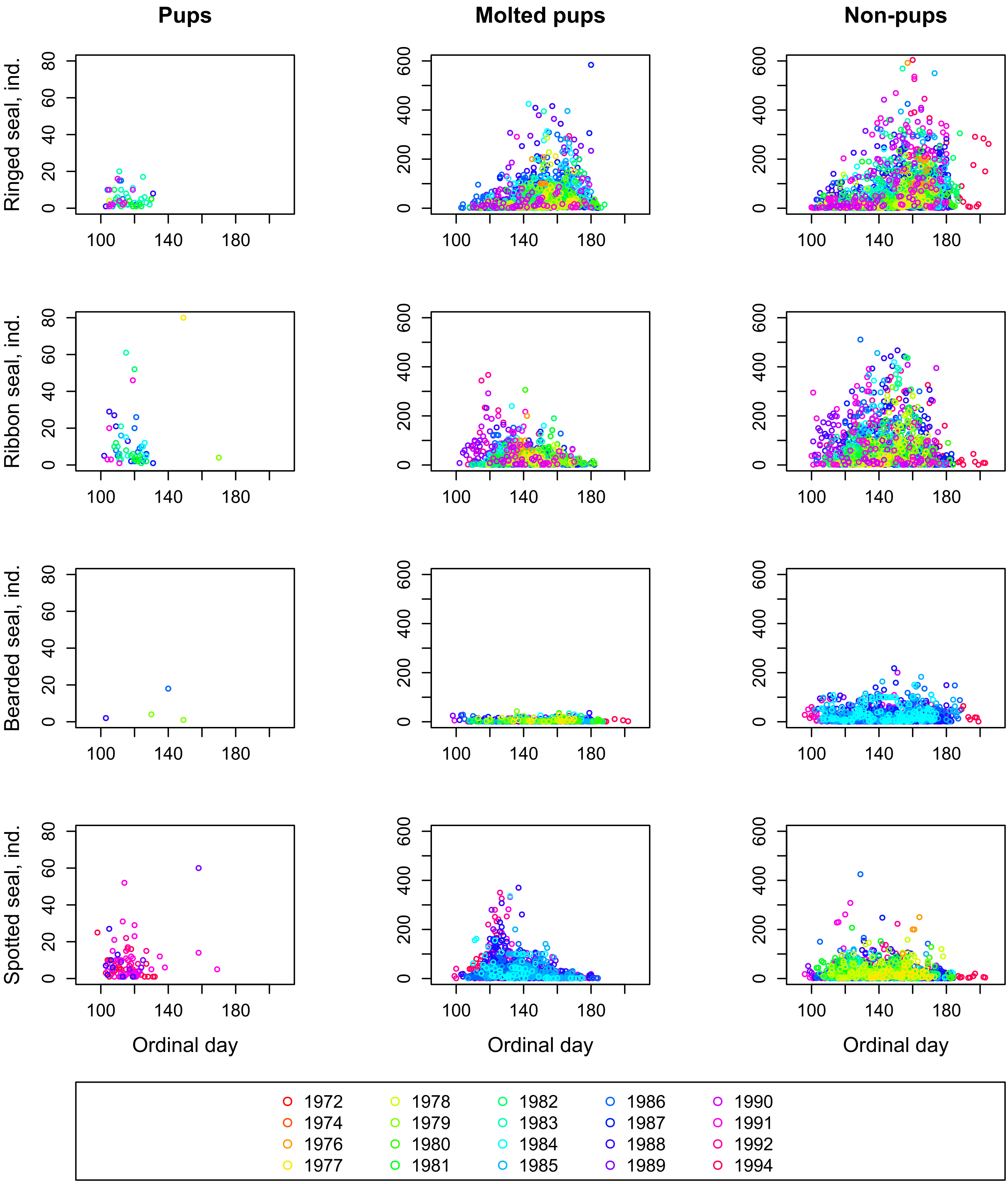

Figure A. Seal harvest timings (ordinal day) and total catches (individuals) by species and age class in the Sea of Okhotsk, 1972-1994. Note that Y-axis scale varies with age class.
