# Supplementary material for: The commercial harvest of ice-associated seals in the Sea of Okhotsk, 1972-1994
Source: PLoS One. 2017 Aug 10;12(8):e0182725. doi: 10.1371/journal.pone.0182725 (PMC5552157; doi:10.1371/journal.pone.0182725)
Supplement: S1 File — (PDF) [file pone.0182725.s004.pdf]

S1 File: Seal harvest distribution maps by month and species in the Sea of Okhotsk, 1972-1994.
Note that total catch scale differs.

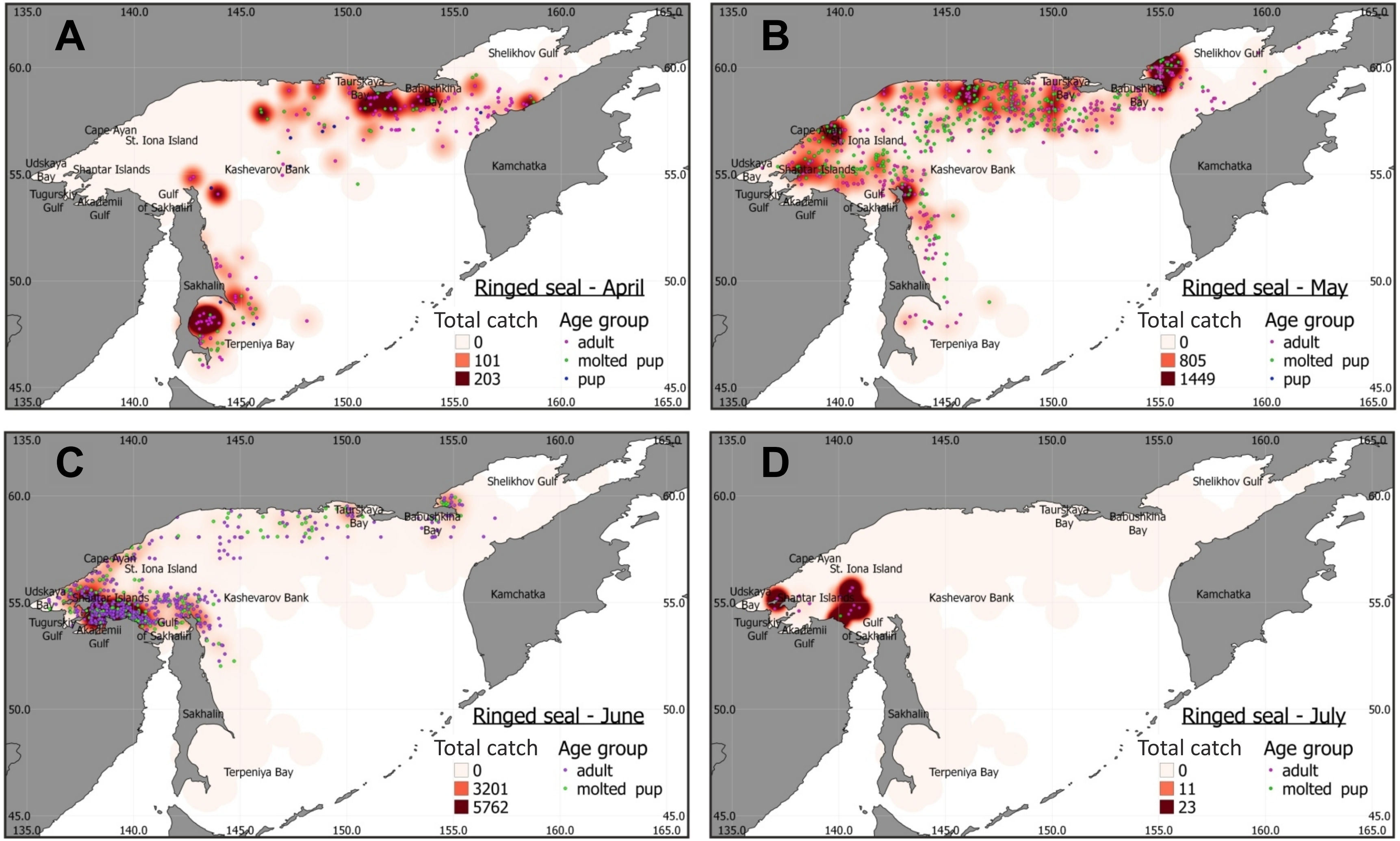

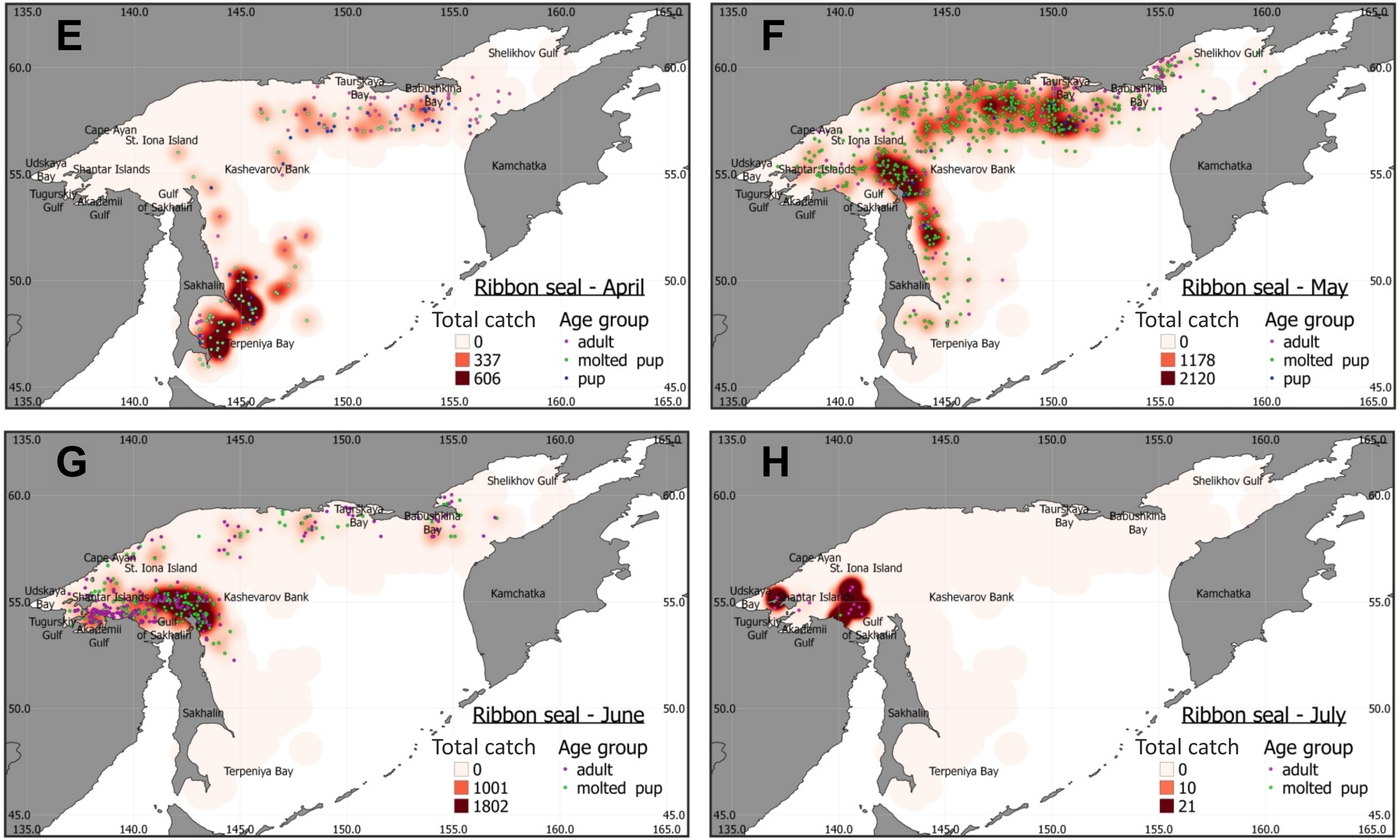
E
Ribbon seal - April
Total catch
0
337
606
Age group
adult
molted pup
pup
F
Ribbon seal - May
Total catch
0
1178
2120
Age group
adult
molted pup
pup
G
Ribbon seal - June
Total catch
0
1001
1802
Age group
adult
molted pup
H
Ribbon seal - July
Total catch
0
10
21
Age group
adult
molted pup
Shelikhov Gulf
Taurskaya Bay
Babushkina Bay
Cape Ayan
St. Iona Island
Udskaya Bay
Shantar Islands
Kashevarov Bank
Kamchatka
Tugurskiy Gulf
Akademii Gulf
Gulf of Sakhalin
Sakhalin
Terpeniya Bay
135.0
140.0
145.0
150.0
155.0
160.0
165.0
60.0
55.0
50.0
45.0

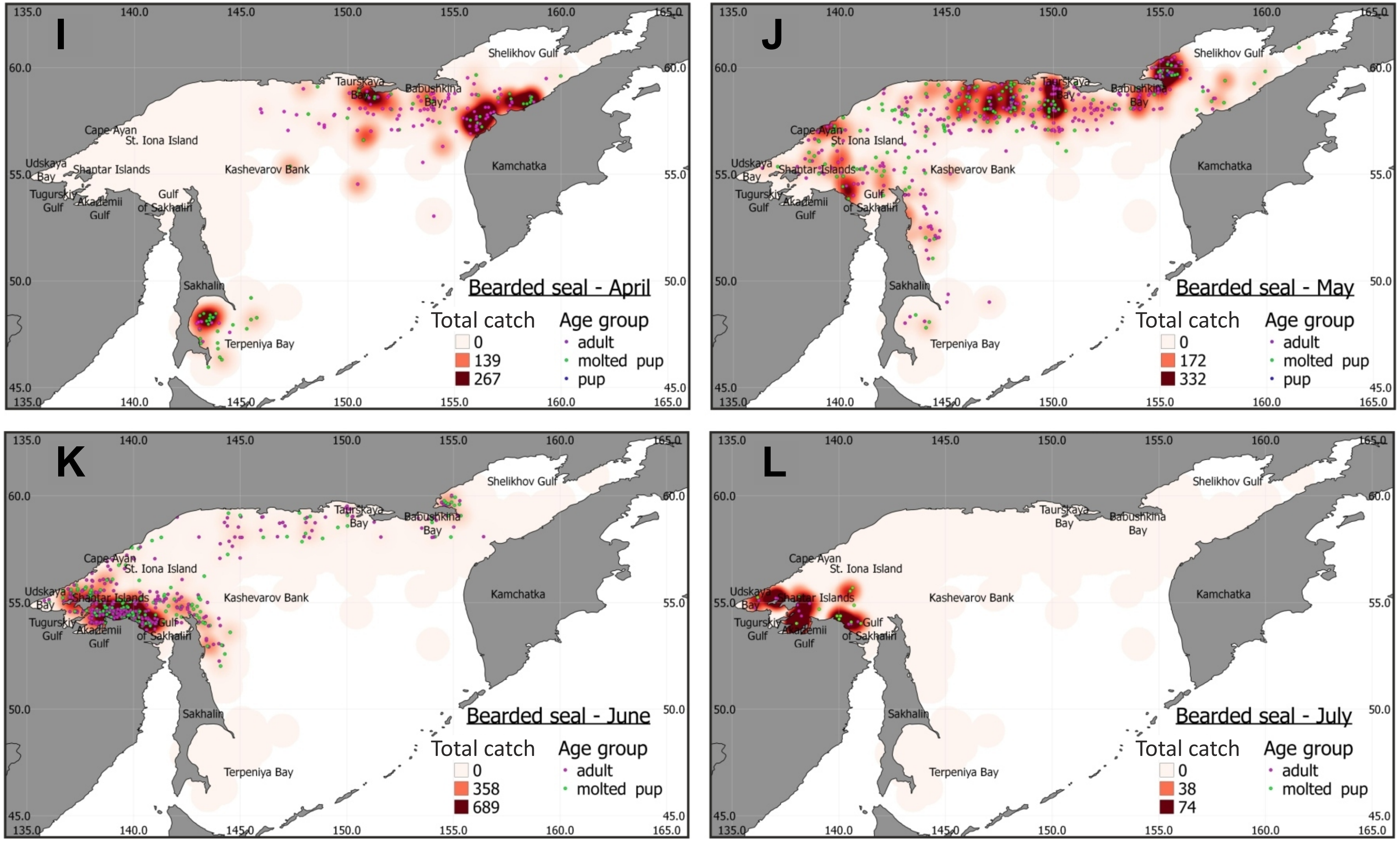

I
Bearded seal - April
Total catch
0
139
267
Age group
adult
molted pup
pup
J
Bearded seal - May
Total catch
0
172
332
Age group
adult
molted pup
pup
K
Bearded seal - June
Total catch
0
358
689
Age group
adult
molted pup
L
Bearded seal - July
Total catch
0
38
74
Age group
adult
molted pup
Shelikhov Gulf
Tauiskaya Bay
Babushkina Bay
Cape Ayan
St. Iona Island
Udskaya Bay
Shantar Islands
Kashevarov Bank
Kamchatka
Tugurskiy Gulf
Akademii Gulf
Gulf of Sakhalin
Sakhalin
Terpeniya Bay
135.0
140.0
145.0
150.0
155.0
160.0
165.0
45.0
50.0
55.0
60.0

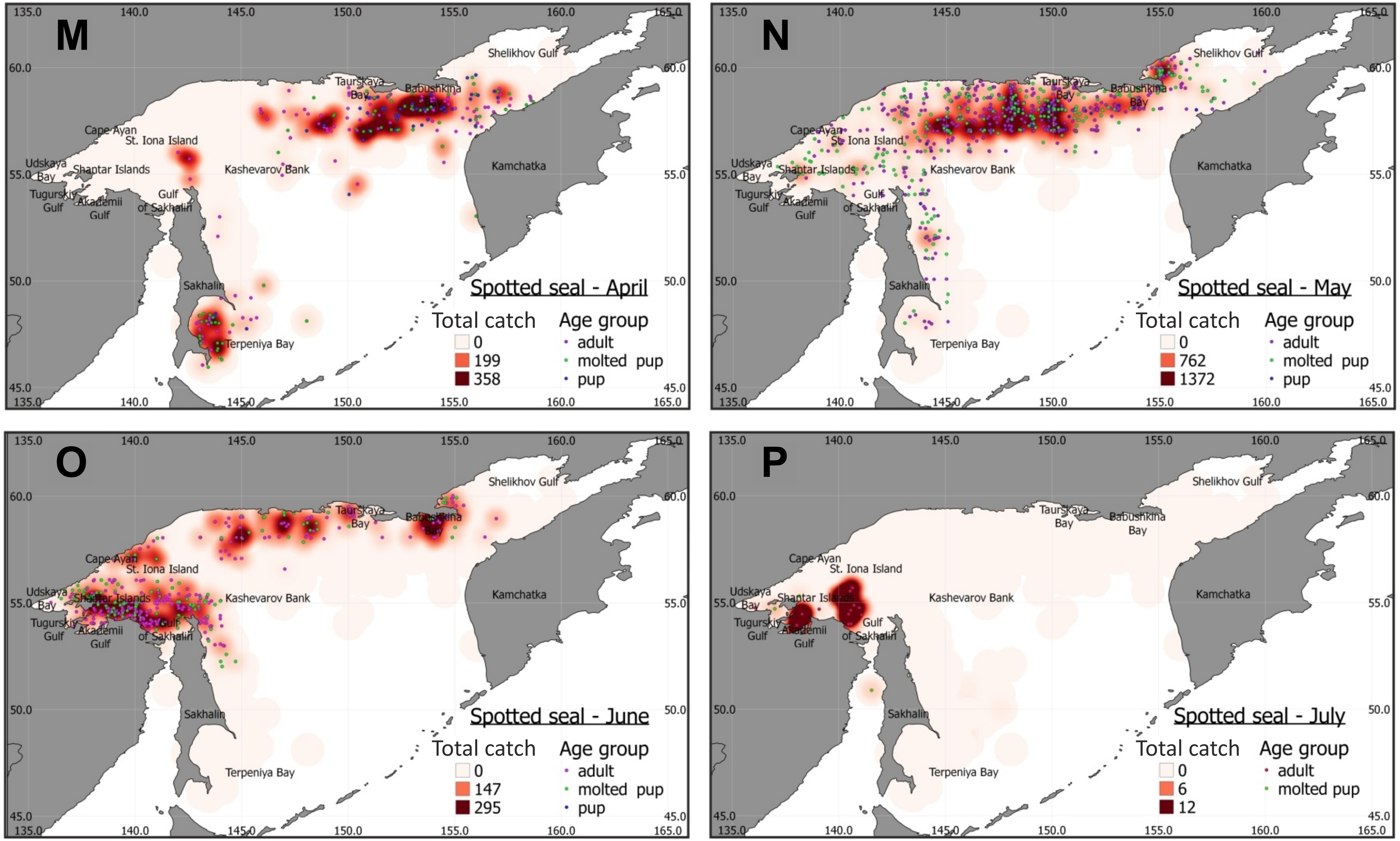
M
Spotted seal - April
Total catch
0
199
358
Age group
adult
molted pup
pup
N
Spotted seal - May
Total catch
0
762
1372
Age group
adult
molted pup
pup
O
Spotted seal - June
Total catch
0
147
295
Age group
adult
molted pup
pup
P
Spotted seal - July
Total catch
0
6
12
Age group
adult
molted pup
Shelikhov Gulf
Taurskaya Bay
Babushkina Bay
Cape Ayan
St. Iona Island
Udskaya Bay
Shantar Islands
Kashevarov Bank
Kamchatka
Tugurskiy Gulf
Akademii Gulf
Gulf of Sakhalin
Sakhalin
Terpeniya Bay
135.0
140.0
145.0
150.0
155.0
160.0
165.0
60.0
55.0
50.0
45.0
